# Impact of Ninjin’yoeito on frailty and short life in klotho-hypomorphic (kl/kl) mice

**DOI:** 10.3389/fphar.2022.973897

**Published:** 2022-10-24

**Authors:** Haruka Amitani, Shigeki Chiba, Marie Amitani, Seiwa Michihara, Risa Takemoto, Likun Han, Nina Fujita, Ryuji Takahashi, Akio Inui

**Affiliations:** ^1^ Pharmacological Department of Herbal Medicine, Kagoshima University Graduate School of Medical and Dental Sciences, Kagoshima, Japan; ^2^ Division of Psychosomatic Internal Medicine, Kagoshima University Graduate School of Medical and Dental Sciences, Kagoshima, Japan; ^3^ Kampo Research Laboratories, Kracie Pharma, Ltd., Tokyo, Japan; ^4^ Education Center for Doctors in Remote Islands and Rural Areas, Kagoshima University Graduate School of Medical and Dental Sciences, Kagoshima, Japan; ^5^ Division of Community-Based Medicine, Kagoshima University Graduate School of Medical and Dental Sciences, Kagoshima, Japan

**Keywords:** Ninjin’yoeito, aging, klotho-hypomorphic mice, frailty, survival, gait, muscle atrophy, telomere

## Abstract

With the recent aging of society, the prevention of frailty has become an important issue because people desire both a long and healthy lifespan. Klotho-hypomorphic (kl/kl) mice are known to show phenotypes of premature aging. Ninjin’yoeito (NYT) is a traditional Japanese Kampo medicine used to treat patients with vulnerable constitution, fatigue or physical exhaustion caused by aging and illness. Recent studies have reported the potential efficacy of NYT against frailty. We therefore evaluated the effect of NYT on the gait function, activity, the histopathological status of organs and survival using kl/kl mice as a model of aging-related frailty. Two sets of 28-day-old male kl/kl mice were assigned to the vehicle (non-treated; NT), 3% or 5% NYT dietary groups. One set of groups (NT, n = 18; 3% NYT, n = 11; 5% NYT, n = 11) was subjected to the analysis of free walking, rotarod, and spontaneous activity tests at approximately 58 days old. Thereafter, we measured triceps surae muscles weight and myofiber cross-sectional area (CSA), and quantified its telomere content. In addition, we evaluated bone strength and performed histopathological examinations of organs. Survival was measured in the second set of groups (NT, 3% NYT and 5% NYT group, n = 8 each). In the walking test, several indicators such as gait velocity were improved in the NYT 3% group. Similar results were obtained for the latency to fall in the rotarod test and spontaneous motor activity. Triceps muscle mass, CSA and its telomere content were significantly improved in the NYT 3% group. Bone density, pulmonary alveolus destruction and testicular atrophy were also significantly improved in the NYT 3% group. Survival rate and body weight were both significantly improved in the NYT3% group compared with those in the NT group. Continuous administration of NYT from the early stage of aging improved not only gait performance, but also the survival in the aging-related frailty model. This effect may be associated with the improvements in aging-related organ changes such as muscle atrophy. Intervention with NYT against the progression of frailty may contribute to a longer, healthier life span among the elderly individuals.

## Introduction

The lifespan of humans has increased in conjunction with the development of modern medicine since the 1950s, and both the share and raw number of older individuals in the total population are growing rapidly (Rau et al., 2008). According to a United Nations report in 2020, the number of persons aged 65 years or over around the world was projected to reach 727 million in 2020 and to more than double by 2050, reaching over 1.5 billion (United Nations, 2020). With this recent aging of society, extending the period during which people can live without having daily life limited by health problems, the “healthy lifespan”, is extremely important to reduce medical and nursing care costs and enrich quality of life for individuals.

Frailty has become the greatest barrier to keep older adults healthy and prolong healthy lifespan. Frailty has been defined as a state of increased physiological vulnerability and is associated with increased risks of negative health outcomes (i.e., morbidity, falls, hospitalization, long-term care, institutionalization, and mortality), causing huge burdens on healthcare and social systems (Clegg et al., 2013; Chen et al., 2014; Cesari et al., 2016). Frailty may represent a transition phase between successful aging and disability. With the gradually increasing number of elderly, global awareness of the need to prevent and delay the onset of frailty is growing (Cesari et al., 2016; Puts et al., 2017).

Frailty, also known as “accelerated aging”, is a multi-factorial phenomenon with a pathological basis in phenomena such as deoxyribonucleic acid (DNA) damage, alterations in gene and non-coding ribonucleic acid (RNA) expression, loss of proteostasis, oxidative stress, and chromatin disruption (López-Otín et al., 2013). Mutations in the klotho can produce a syndrome that seems to considerably accelerate aging (Sasaki et al., 2013). Klotho is a type I single-pass transmembrane protein with beta-glucuronidase activity that has been implicated in influencing the aging process (Tohyama et al., 2004). Levels of Klotho protein decline with normal aging in mice and in humans (Arking et al., 2002). Klotho-hypomorphic (kl/kl) mice are known for showing premature aging phenotypes, including a short lifespan of 8–9 weeks and several conditions that closely resemble those found in human aging-related frailty, such as weight loss, hypokinesis, gait disturbance, osteoporosis, and fat and muscle atrophy (Kuro-o et al., 1997; Iida et al., 2011).

Ninjin’yoeito (NYT), a traditional Japanese Kampo medicine, is used to treat patients with vulnerable constitution, fatigue or physical exhaustion caused by aging and during/after an illness, loss of appetite, persistent cough, cold limbs, anemia, and mental disequilibrium (Uto et al., 2018). NYT comprises 12 herbs: peony root, Japanese angelica root, *Citrus unshiu* peel, astragalus root, cinnamon bark, ginseng, atractylodes rhizome, *glycyrrhiza*, rehmannia root, schisandra fruit, poria sclerotium, and polygala root. Basic research has reported the effect of the respective herb components, such as improvement of appetite loss and sarcopenia with hesperidin in *Citrus unshiu* peel (Fujitsuka et al., 2016), enhanced insulin sensitivity and prevention of atherosclerosis with astragalus root (Chen et al., 2012), and prevention of neurodegeneration with ginseng (Suzuki et al., 2015). In addition, several recent review articles have reported the potential efficacy of NYT against frailty (Miyano et al., 2018; Uto et al., 2018; Takayama et al., 2019).

The present study therefore evaluated the effect of NYT on survival, the histomorphometric status of organs, gait function and activity using kl/kl mice as a model of aging-related frailty.

## Materials and methods

### Animals and animal care

We purchased 28-day-old male kl/kl mice and litter-mate wild-type control mice (WT) with a C3H/J x C57BL/6J mixed background (Iida et al., 2011) from CLEA Japan Inc. (Osaka, Japan). Paired WT and kl/kl mice were housed in one cage with an air filter cover and bred in clean-shelf (Natsume Seisakusho Co., Tokyo, Japan) because kl/kl mice are extremely vulnerable to hypothermia, infection, and social isolation stress. Room temperature was controlled to 26 ± 1°C and humidity was 50–60%, with a daily 12:12 h light/dark cycle. Mice were fed pasteurized moderate-fat (MF) rodent diet (Oriental Bio Co., Tokyo, Japan) mixed with vehicle or 3% or 5% (w/w) NYT (NT or 3% NYT or 5% NYT) that was placed on the bottom of the cage. Water was available *ad libitum*. All experimental protocols were approved by the Animal Care and Use Committee of Kracie Pharm Co. Ltd.

### Reagent

NYT (lot. 15112017) was produced and provided by Kracie Pharma, Co. Ltd.; detailed properties have been described in a previous study (Murata et al., 2018).

### Survival period measurement

Eighteen kl/kl mice each were divided into 3 groups, and fed MF vehicle (non-treated; NT), 3%, or 5% (w/w) NYT diet until all kl/kl mice died. The surviving individuals were checked every morning and evening. The kl/kl mice were purchased by selecting mice with a total body weight of 8.0 ± 1.0 g at 4 weeks old. The body weights of WT and kl/kl mice were recorded every 6 days from 28 days old.

### Tissue processing

At dissection (58–60 days), Mice were perfusion-fixed in 4% paraformaldehyde for 10 min, and tissues such as heart, lung, testes, thymus, spleen, and gastrocnemius and soleus muscles from the triceps surae muscle of the lower legs were collected, washed with saline, and weighed. Images of all tissues were then taken. Weights were normalized to body weight. The organs were then sliced at a thickness of 5 mm and fixed in 4% paraformaldehyde/phosphate-buffered saline (PBS) for 48 h and stored in 50% ethanol/PBS (-) at 4°C.

### Evaluation of alveolar condition

After acquiring 8 images of each stained sample using an (Axio Observer Z1; Carl Zeiss, Jena, DE) optical microscope at ×100, the mean linear intercept (Lm) of the alveolar wall was calculated (Munoz-Barrutia et al., 2012). In summary, 20 thin lines were drawn at equal intervals on each image, and the length of the thin line (l) and the number of intersections with the alveoli (Si) were measured using ImageJ Fiji analytical software (https://imagej.net/software/fiji). Lm was calculated using the following formula. Lm = number of fine lines (h) × length of fine lines (l)/number of intersections with alveoli (Si). The average of 8 images was used as the Lm for each individual.

### Counting the number of sperm in the seminiferous tubules

After fixation with 4% paraformaldehyde (PFA), testes were subjected to preparation in paraffin-embedded sections followed by staining with hematoxylin and eosin. Section images were captured using an optical microscope at 600× for 10 images per sample. The number of sperm per 5 mm^2^ in the seminal duct was then manually counted.

### Bone radiographic examination

The tibiae of the hind limb were harvested, immersion-fixed for an additional 72 h, and stored at 4°C in 50% ethanol. Tibiae were scanned using a Skyscan 1174 micro-computed tomography (CT) scanner (Bruker, Billerica, MA, United States) with a pixel resolution of 9.5 μm, an X-ray tube voltage of 50 kV, an X-ray tube current of 800 μA, a 0.5 mm aluminum filter, and a rotation angle of 0.8°. The acquired data were reconstructed on NRecon software and analyzed with the 3-dimensional CT Analyzer software (Bruker). Cortical bone was measured using a 0.4 mm area in the distal direction starting 1.7 mm below the lower edge of the growth plate as the measurement area, and bone area, bone thickness, porosity, and polar moment of inertia item were measured. For analysis of trabecular bone, the 1.7 mm area distal to the lower edge of the growth plate was used as the measurement area, and tissue volume, trabecular number, and trabecular separation were measured.

### Immunoblotting

After frozen gastrocnemius muscle was crushed using Tissue Lyser (QIAGEN, Venlo, NLD), proteins were extracted using radioimmunoprecipitation assay (RIPA) buffer with 1% protease and a phosphatase inhibitor (Nacalai Tesque, Tokyo, Japan), separated by sodium dodecyl sulfate-polyacrylamide gel electrophoresis (SDS-PAGE) at 10 µg/lane, and transferred to polyvinylidene (PVDF) membranes (BIORAD, CA, United States). After blocking with 5% skim milk for 1 h, membranes were incubated with the primary antibodies anti-4E-BP1(pSer65) Ab (#9451 Cell Signaling Technology (CST), MA, United States), anti-Phospho-4EBP-1(Thr37/46)Ab (#9459; CST), anti-atrogin-l Ab (#9452; CST), anti-glyceraldehyde-3-phosphate dehydrogenase (GAPDH) Ab (#G8795; Sigma-Aldrich, MO, United States), anti-forkhead box O (FoxO)-1(pSer256) Ab (#g234; CST), anti-FoxO-1 Ab (#2880; CST), anti-Akt (pSer473) Ab (#4051; CST), anti-Akt1 Ab (#9272; CST), anti-muscle RING-finger protein (MURF)-1 Ab (#MP3401; ECM bioscience, KY, United States), anti-peroxisome proliferator-activated receptor gamma coactivator 1 (PGC1) Ab (ab54481; Abcam, Cambridge, United Kingdom), anti-p70S6K Ab (#9234; CST), anti-p70S6K Ab (#2708; CST), anti-light chain 3B (LC3B) Ab (#L7543; Sigma) in 5% BSA/TBS for 8 h at 4°C. Secondary antibodies of anti-rabbit IgG-HRP (RS3251; GE Healthcare, IL, United States) or anti-mouse IgG antibody (#7076, CST) were reacted for 1 h at room temperature. Proteins were then detected by LAS-3000 (Fuji Film, Tokyo, Japan) using the ECL Prime Western Blotting Detection Reagent (GE Healthcare). Restore WB Stripping Buffer (Thermo Fisher, MA, United States) was used for re-probing.

### Measurement of muscle mass and cross-sectional area

The triceps surae muscle of the lower limbs comprises the soleus and gastrocnemius muscles, and its activity indirectly controls step length and gait velocity (Honeine et al., 2013; Honeine et al., 2014). Muscular mass of the hind leg was measured at 59 days old, when the body weight of kl/kl mice gradually decreased. The triceps surae muscle was collected from the lower legs; the gastrocnemius and soleus muscles were isolated, and wet weights were measured as described previously (Shinin et al., 2009). Weight of each muscle was normalized to body weight. The cross-sectional area (CSA) were measurement using the paraffin-embedded muscle section. Briefly, fixed sections were blocked with BIOXALL (vector Laboratory, CA, United States) and BlockingOne Hist (Nakarai Tesuque) after antigen retrieval with Immunosaver (Nissin EM Co., Tokyo, Japan). Sections were then incubated with anti-dystrophin Ab (ab15277, Abcam) for 3 h at room temperature, incubated with anti-Rabbit 2nd Ab POD-Conjugate (Takara, Shiga, Japan) for 30 min, developed with Immpact-DAB (Vector Lab), then stained with hematoxylin. Images of sections were acquired using optical microscope with ×200 tiling mode. The thickness of myofibers in muscle and its strength are linearly correlated, and the aging reduces the myofiber CSA (Lexell et al., 1988). To determine the CSA of muscle fibers, the perimeter of the area surrounded by dystrophin was enclosed and the internal area was measured as CSA using ImageJ Fiji, described previously (Mayeuf-Louchart et al., 2018). Muscle fibers suitable for measurement were selected those that were completely surrounded by a cell membrane and free of distortion, scarring, or bending. Elongated fibers showing an oblique cross-section were also excluded.

### Determine the fast/slow myofiber and measurement of its CSA

Paraffin-embedded sections of soleus muscle were subjected to antigen retrieval with Immunosaver (Nissin EM), followed by blocking with BIOXALL (vector lab) and Blocking Reagent A (POD Conjugate Set Anti Mouse for Mouse Tissue; Takara Bio). Sections were incubated with anti-slow myosin skeletal muscle Ab (Abcam) for 2 h at room temperature, blocked with Blocking Reagent B (Takara bio) for 10 min, incubated with POD conjugate anti-mouse for mouse tissue (Takara bio) for 30 min and developed with Immpact NovaRed (Vector Lab). Further, after treatment with 10% acetic acid at 37°C for 30 min, blocking was again with Blocking Reagent A and Fab Anti-Mouse IgG (abcam) for 60 min at room temperature. Next, sections were incubated with anti-fast myosin skeletal muscle Ab (Abcam) for 2 h at room temperature, blocked with Blocking Reagent B for 10 min, and incubated with POD Conjugate anti-mouse for mouse tissue (Takara) for 60 min, then developed with HistGreen (Eurobio, Paris, France) and stained with hematoxylin. Stained sections were captured using the optical microscope in ×200 tiling mode. After extracting color regions similar to Red or Green from the section image using Paintshop Pro (Corel, Ottawa, CA), the CSA and number of regions above a certain size were measured using ImageJ Fiji.

### Quantification of telomere content

To examine the degree of aging of lower limb muscles, the telomere content of genomic DNA was evaluated. Genomic DNA was isolated from these muscles using a Wizard Genomic DNA Purification Kit (Promega, WI, United States) in according with the instructions. DNA was subjected to quantification of relative telomere copy number or a single copy reference gene region: 36B4 by qPCR as previously described previously (Callicott and Womack, 2006) using Power-Syber green master mix (Thermo Scientific) on a StepOnePlus Realtime thermal cycler (Thermo Scientific).

### Immunohistochemical detection of oxidative DNA damage

Thin sections of deparaffinized gastrocnemius muscle were treated with immunosaver (Nisshin EM) for antigen retrieval and then with streptavidin and biotin blocking solution (Vector Labs.) and 5% BSA for blocking of tissue. In addition, sections were treated with biotinylated anti-DNA/RNA damage (8-OHdG) antibody [15A3] (Abcam), followed by streptavidin alkaline phosphatase (Vector Labs.). The oxidized nuclei were visualized with ImmPACT Vector Red (Vector Labs.) and contrast staining with hematoxylin. Tissue images were obtained using optical microscope with ×100 tiling mode. After excluding stromal tissue, blood vessels, and blood cells from the images, the number of nuclei clearly identified as 8-OHdG positive (+) and total nuclei were counted using ImageJ Fiji.

### Walking test

To measure walking performance, a rectangular parallel epiped walking path, 1200 mm (L) × 40 mm (W) × 15 mm (H) was built using transparent acryl, and the open end of the path was connected to a dark room. Mice at 58 days old were placed in the dark room, acclimatized for 15 min, then placed into the apparatus from the opposite side of the dark room; mice were allowed to walk through the path with video tracking of under and side views at 60 frames/s. The trial was performed three times with a 15-min interval between consecutive trials. Video footage in which mice trotted, bounded, or galloped was not adopted for test data and retesting was performed (Bellardita and Kiehn, 2015).

Mobility impairment in the elderly is reflected in the pathognomonic parameters of walking (Pirker and Katzenschlager, 2017). Gait velocity is an important and representative index for estimating walking performance. Gait velocity was measured from the time and distance of the section where mice walked continuously. Wild-type and kl/kl mice show significant differences in physique, so gait velocity was corrected by the body length (length from the base of the tail to the neck). Stride time/step was measured manually as the amount of time between two initial paw contacts with the walking path for the same paw from a series of gait images divided into 1/60th of a second. Temporal asymmetry was calculated as follows: |time of right foot strike–time of left foot strike|/stride time, referred to as temporal gait asymmetry (Jacobs et al., 2014) and describes the synchronicity of the left-to-right foot-strike sequence. To obtain footprint images, the sole of each foot of the mouse was painted with non-toxic green color ink and dried; mice were then allowed to walk, and images of individual paws in contact with the walking path were extracted from the movie using ImageJ Fiji.

To discriminate between the forelimb and hind-limb, the image of the fore sole was colored green, that of the hind sole was colored red, and images were merged. Stride length, stance, step width were measured as described previously (D'Hooge et al., 1999). Representative values for each parameter were calculated using the average of consecutive strides for each of the four paws. For group comparisons, the right and left paws were averaged to give representative values for the fore-paws and hind-paws for each mouse.

### Rotarod test

To evaluate motor coordination, balance, grip strength, and motor learning ability for smoothly walking, the rotarod test was performed using a rotarod apparatus MK-630B (Muromachi Kikai, Tokyo, Japan) according to the instructions. Briefly, animals were placed on a rotating cylinder, and a training session was performed at a constant speed of 4 rpm for 1 min. Mice were then subjected to the walking test on the accelerating spindle (4–20 rpm over 180 s), and the latency for mice falling off the cylinder was recorded. Mice that rotated passively were recorded as having fallen. The trial was performed three times with a 30-min interval between consecutive trials. Mean times of the test were recorded for each animal.

### Spontaneous locomotion

In kl/kl mice, spontaneous motor activity was decreased due to the progression of frailty, indicated by decreased physical ability and strength (Kuro-o et al., 1997). Two types of spontaneous locomotion activity tests were performed. First, total distance traveled and resting time in the open field test were used as short-term locomotor activities in kl/kl mice (Kuro-o et al., 1997; Eren et al., 2014; Seibenhener and Wooten, 2015). Briefly, all mice were transported to the test room and left undisturbed for at least 30 min before tests started. Each mouse was placed on the center of an opaque open field apparatus [300 mm (W) × 300 mm (L) × 300 mm (H)] and was allowed to explore freely; movement was recorded with a video camera from above for 10 min. The total distance traveled and resting time were measured using ANY-maze Video Tracking Software (Stoelting Co., IL, United States). The longest travel distance out of three trials was subjected to the statistical analysis.

Second, an overnight locomotor test was performed in the breeding cage to account for the possibility that locomotor impairment was limited to conditions when the mice were temporarily placed in a new environment. For long-term testing, two mice of the same type were put in one breeding cage, and locomotor activity was recorded with an infrared sensor (Supermex apparatus; Muromachi Kikai, Tokyo, Japan) for 12 h during the nocturnal period according to the instructions. Mice were provided ad labium access to diet water.

### Grip strength test

Grip strength of all limbs was measured using a digital force meter (MK-380M; Muromachi Machinery Co., Ltd.). The mouse was brought down vertically onto a metal mesh attached to the force meter and allowed to grip the metal mesh, then was gently pulled away at a constant speed until grip was released. Peak tension (grams force) on release of the grip was recorded as the grip force. The grip force of each mouse was measured four times with a 5-min interval between measurements, and the highest value was adopted.

### Statistical analyses

All data are expressed as mean ± standard error of the mean. Statistical comparisons were performed using one-way ANOVA for parametric tests followed by Dunnett’s test or Kruskal–Wallis of variance for non-parametric tests followed by Steel’s test. Overall and median survival were determined using Kaplan-Meier analyses and log-rank testing of variance. Differences showing values of *p* < 0.05 were considered statistically significant, as represented by a ‘*’ sign, and *p* < 0.01 is represented as a ‘**’ sign on the graph. All analyses were performed using the R statistical platform (ver. 3.4.2; https://www.r-project.org/).

## Results

### Survival time and weight remained unchanged in the NYT group during the terminal phase

**FIGURE 1:**
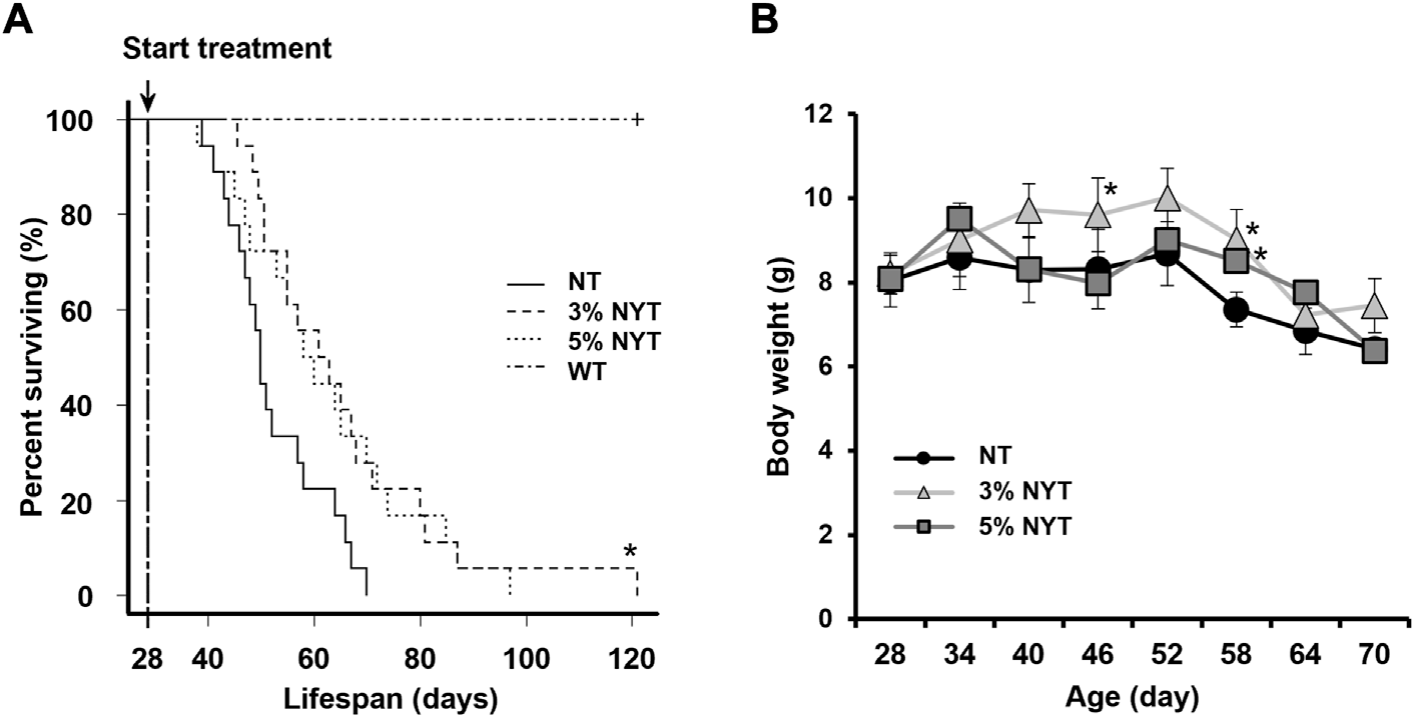
Survival rate and weight change with aging. Survival curves of Kaplan-Meier analysis are shown **(A)**. Treatment was started when the mice were 28 days old. Log-rank test was employed to detect significant differences (*p < 0.05, Bonferroni comparison, n = 18) Body weight **(B)** was measured every 6 days and is shown. Data are plotted as mean and SEM values. Significant differences are presented on histograms: (*p < 0.05, Dunnett’s contrast).

Data regarding changes in survival are shown in Figure 1A. In the NT group, median survival was 50 days, considerably shorter than that in the WT group (>120 days) (Kuro-o et al., 1997). In contrast, median survival was 62 days for the 3% NYT group and 59 days for the 5% NYT group. A significant difference in multiple comparisons was observed among all groups (*p* < 0.001). In addition, when groups were compared, the 3% NYT group showed significantly longer survival than the NT group (*p* = 0.024). However, duration for the 5% NYT group was not significantly different (*p* = 0.134). No mice in the WT group died during the test period of about 120 days.

Body weight data are shown in Figure 1B. Body weight did not change significantly from 28 to 52 days in the NT group, while a slight increase was observed from 28 days to 40 in the 3% NYT group. Moreover, at 58 days old, when the number of deaths in the NT group increased, 8.39% weight loss was observed compared to the initial body weight. In contrast, 9.79% and 5.22% of the weight gain in the 3%NYT and 5%NYT groups was observed, respectively, and was significantly different from that in the NT group (*p* = 0.003 and *p* = 0.038, respectively).

### Improvement in cardiorespiratory function with NYT treatment

**FIGURE 2:**
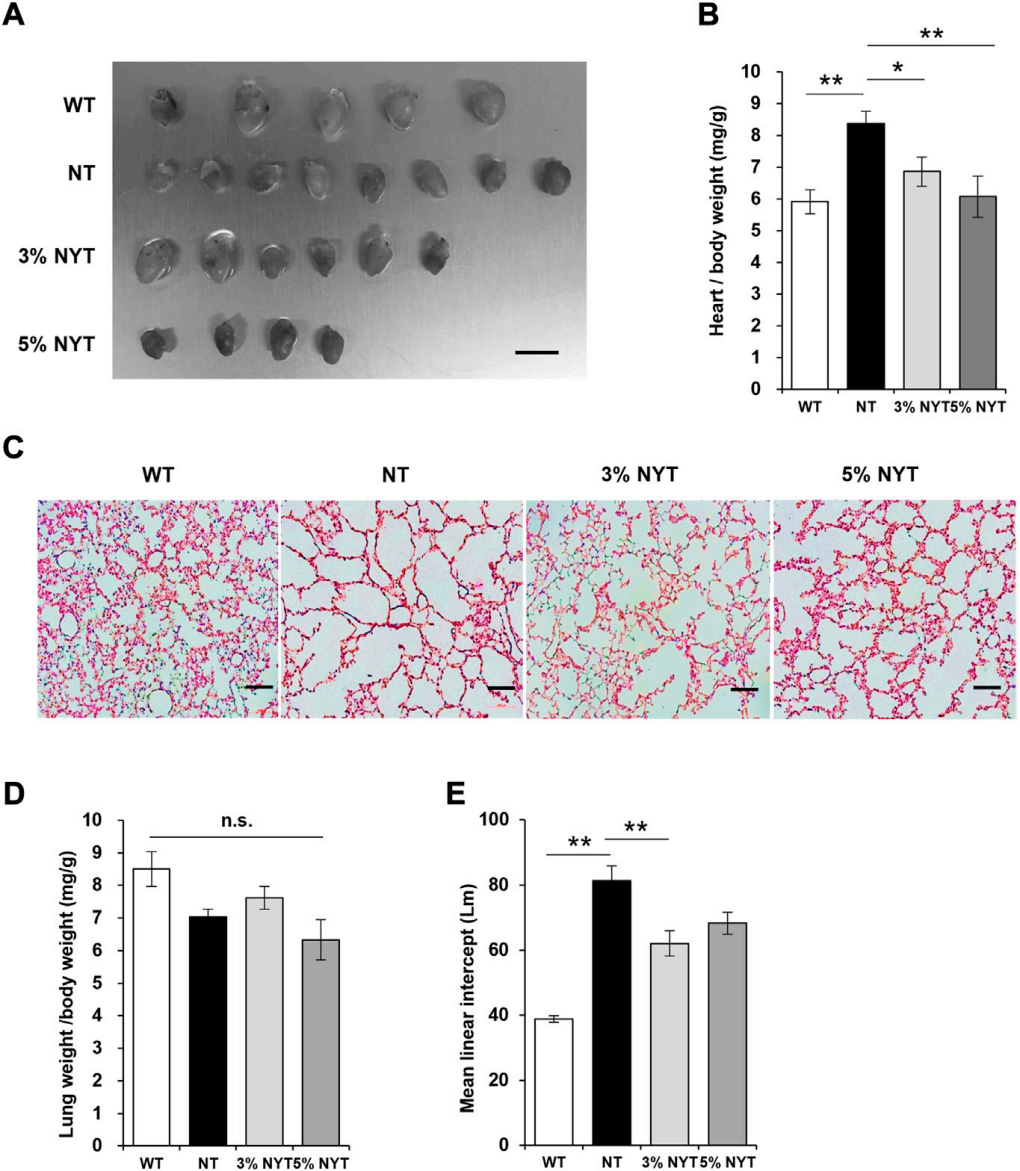
Effect of NYT on Heart weight changes and degree of alveolar damage with aging. The dissected heart tissue images **(A)** on 59 days old and its wet weights **(B)** are shown, scale bar indicated 7 mm. The wet weights **(D)** of lung tissue are shown. Histological staining (hematoxylin/eosin) of lung tissue section are shown in **(C)**, scale bar indicated 100 μm. The degree of alveolar damage was determined by the Mean linear intercept (Lm) method and is shown in **(E)**. Data are indicated as mean and SEM values. Significant differences are presented on histograms: [*p < 0.05, **p < 0.01, Dunnett’s contrast, WT (*n* = 5), NT (8), 3%NYT (6), 5%NYT (4)].

The influence of aging on cardiac hypertrophy is well known, and a similar phenotype has been reported in Klotho deficient mice (Kuro-o et al., 1997; Chen et al., 2022). Tissue images of hearts and the wet weight relative to body weight are shown in Figures 2A,B. The heart weight of NT group was significantly higher than the wild type (*p* = 0.002). In contrast, the heart weight of the 3% NYT and 5% NYT groups were significantly lower than the NT group (*p* = 0.048 and *p* = 0.002, respectively).

Emphysema-like alveolar damage has been observed in aged kl/kl mice (Kuro-o et al., 1997). HE-stained images of lung tissue sections are shown in Figure 2C. Lung wet weight to body weight showed no significant difference between groups (*p* = 0.054) (Figure 2D). Alveoli were more damaged in the NT group than the WT group, and large void spaces were observed in the tissue. In contrast, some alveoli appeared to remain in the 3% NYT group. Therefore, the level of lung damage was measured using the linear intercept (LM) index (Figure 2E). Lm in the NT group was 109.61% elongated and lung damage was more advanced compared to the WT group (*p* < 0.001). In contrast, Lm elongation in the 3% NYT group was maintained at 59.98%, different significantly from that in the NT group (*p* = 0.006).

### Improvement in thymus atrophy and cellular population with NYT treatment

**FIGURE 3:**
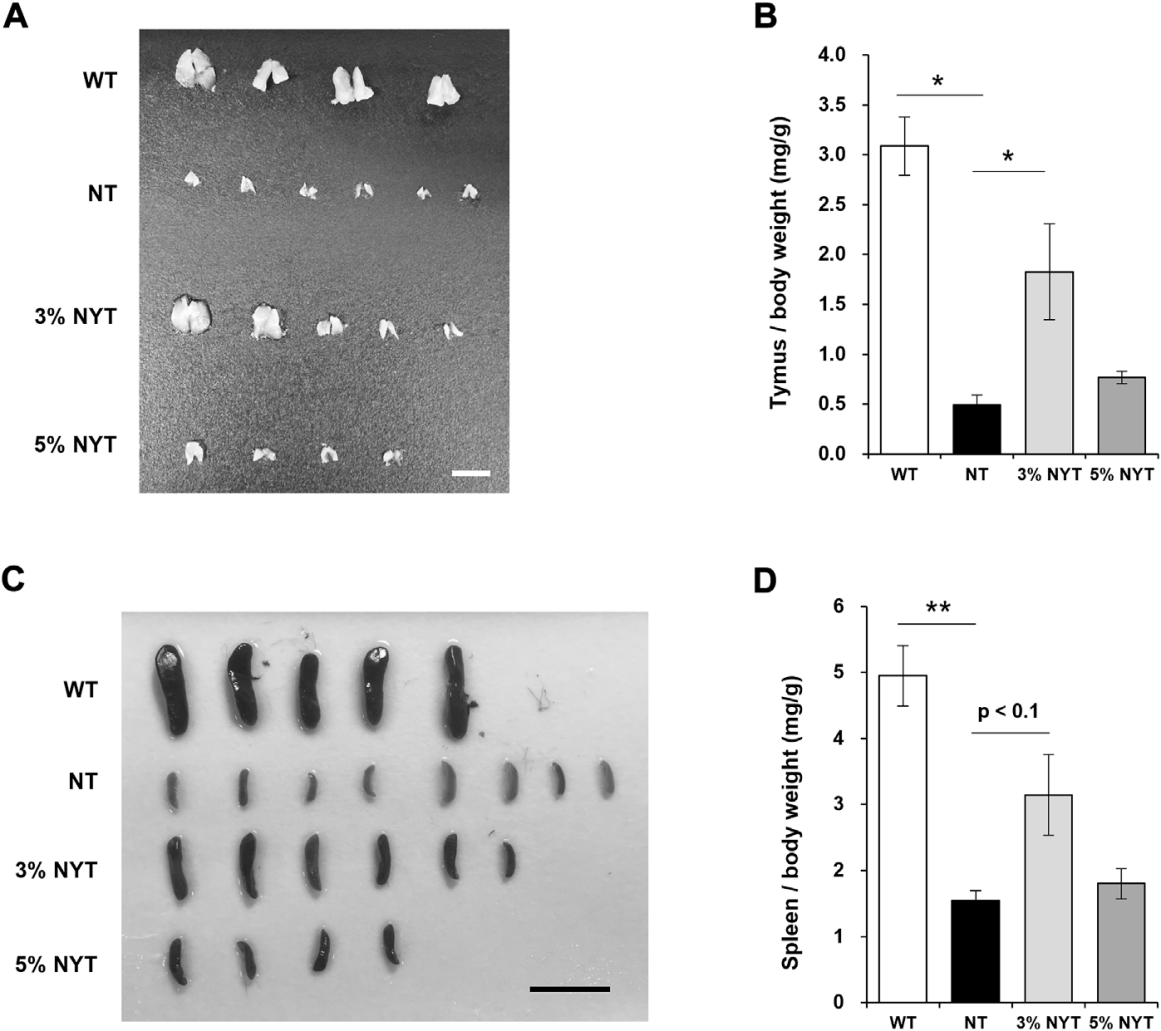
Effect of NYT on age-related splenic and thymic atrophy. The dissected Thymus **(A)** and Spleen **(C)** tissue images on 59 days old and its wet weights **(B**, **D)** are shown, scale bar indicated 5 mm **(A)**, 15 mm **(C)**. The wet weights of lung tissue are shown **(D)**. Data are indicated as mean and SEM values. Significant differences are presented on histograms: [*p < 0.05, **p < 0.01, Steel test **(A)**, WT (*n* = 4), NT (6), 3%NYT (5), 5%NYT (4); Dunnett’s contrast **(B)**, WT (*n* = 5), NT (8), 3%NYT (6), 5%NYT (4)].

Tissue images of the thymus and spleen are shown in Figures 3A,C, and wet weights relative to body weight are shown in Figures 3B,D. Atrophy of the thymus and spleen in aged kl/kl mice has also been reported (Kuro-o et al., 1997). The thymus weight of NT group was significantly lower than the wild type (*p* = 0.029). In contrast, the thymus weight of the 3% NYT groups were significantly higher than the NT group (*p* = 0.018). However, as reported previously (Kuro-o et al., 1997), several of the thymus glands of kl/kl mice were not found at the time of dissection. The spleen weight of NT group was significantly lower than the wild type (*p* < 0.009). In contrast, the spleen weight of the 3% NYT groups were significantly higher than the NT group (*p* = 0.0153).

Since NYT has been reported to be effective in myelosuppression and anemia due to chemotherapy and radiation therapy, and since a trend was seen toward maintenance of the thymus and spleen, the leukocyte population of the peripheral blood was examined for reference (Supplementary Figure S1). Among these, total leukocyte counts were significantly decreased in NT compared to WT, but no significant improvement was evident in the 3% or 5% NYT groups. In contrast, monocytes were significantly maintained in the 3% and 5% NYT groups compared to the NT group (*p* = 0.006 and 0.006, respectively). Similarly, in lymphocytes, significant maintenance was observed in the 3% NYT group and 5% NYT group (*p* = 0.008 and 0.009) and B cells (*p* = 0.008 and 0.009), respectively, compared to the NT group. These data suggest that NYT may affect the cellular population of lymphocytes and monocytes. On the other hand, the granulocyte population was seemed to much higher compare to background strain, and these were treated as reference.

### Improvement in gonadal function with NYT treatment

**FIGURE 4:**
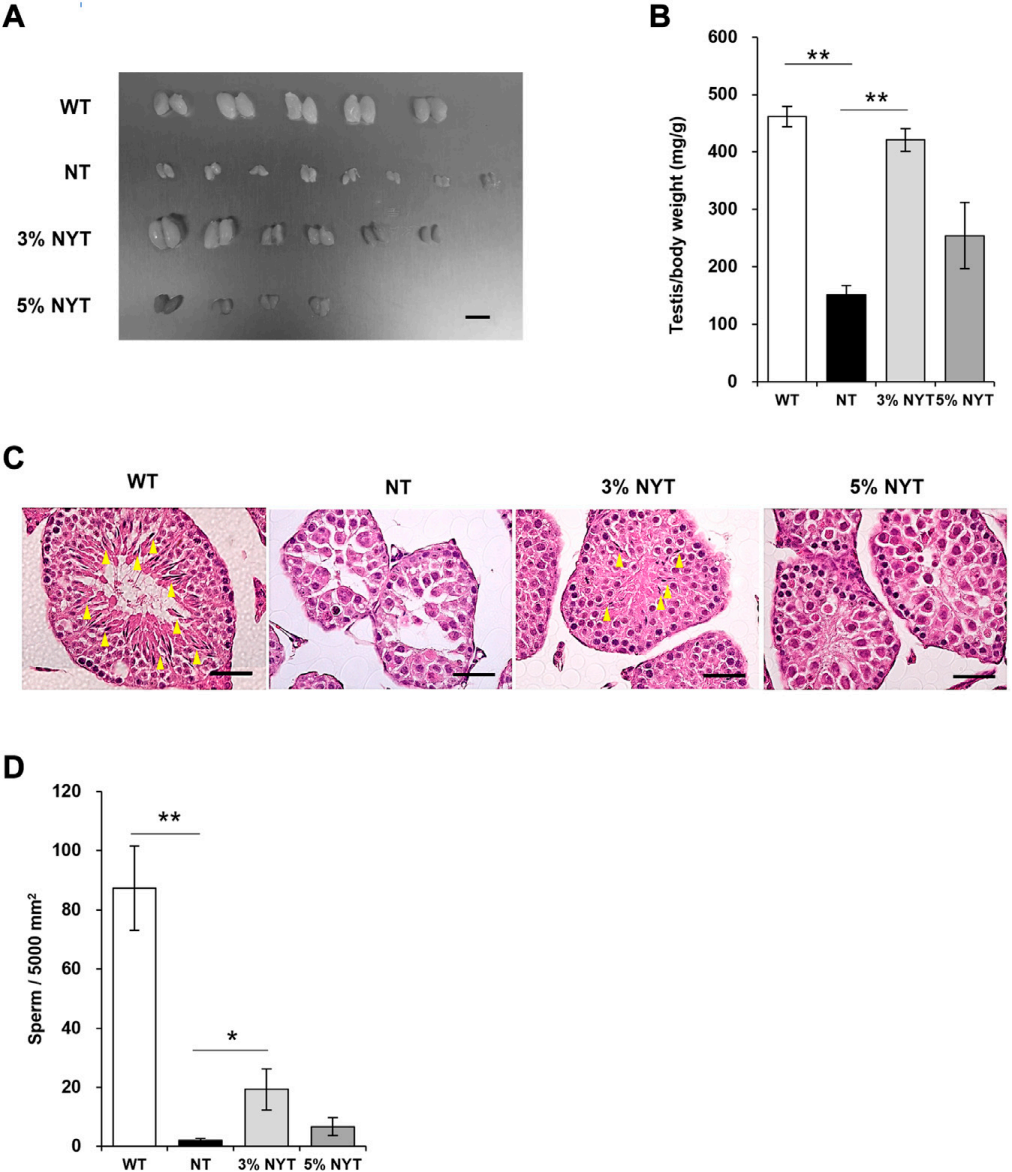
Effect of NYT on suppression of testicular growth and spermatogenesis with premature aging. The dissected Testis tissue images **(A)** on 59 days old and its wet weights **(B)** are shown, scale bar indicated 8 mm **(A)**. Histological staining (hematoxylin/eosin) of testicular tissue are shown in **(C)**, scale bar indicated 40 μm. Yellow arrows indicate spermatozoa in the seminiferous tubules. The number of spermatozoa in the seminiferous tubules of the testicular cross section was counted and are shown in **(D)**. Data are indicated as mean and SEM values. Significant differences are presented on histograms: [*p < 0.05, **p < 0.01, Steel test, WT (*n* = 5), NT (8), 3%NYT (6), 5%NYT (4)].

Tissue images of the testes are shown in Figure 4A, and wet weight relative to body weight is shown in Figure 4B. Stalled development of male and female genitalia and gonads has been reported in kl/kl mice (Kuro-o et al., 1997). The testis weight of NT group was significantly lower than the wild type (*p* = 0.009). In contrast, the testis weight of the 3% NYT groups were significantly higher than the NT group (*p* = 0.006). To assess the maturation level of testicular tissue, sperm-like tissues in the seminiferous tubules were evaluated (Figure 4C). Sperm-like tissue was decreased 97.75% in the NT group compared to WT group (*p* = 0.009) (Figure 4D). In contrast, the 3% NYT group maintained a 77.93% decreased, significantly different from that in the NT group (*p* = 0.013).

### Improvement in osteoporosis with NYT treatment

**FIGURE 5:**
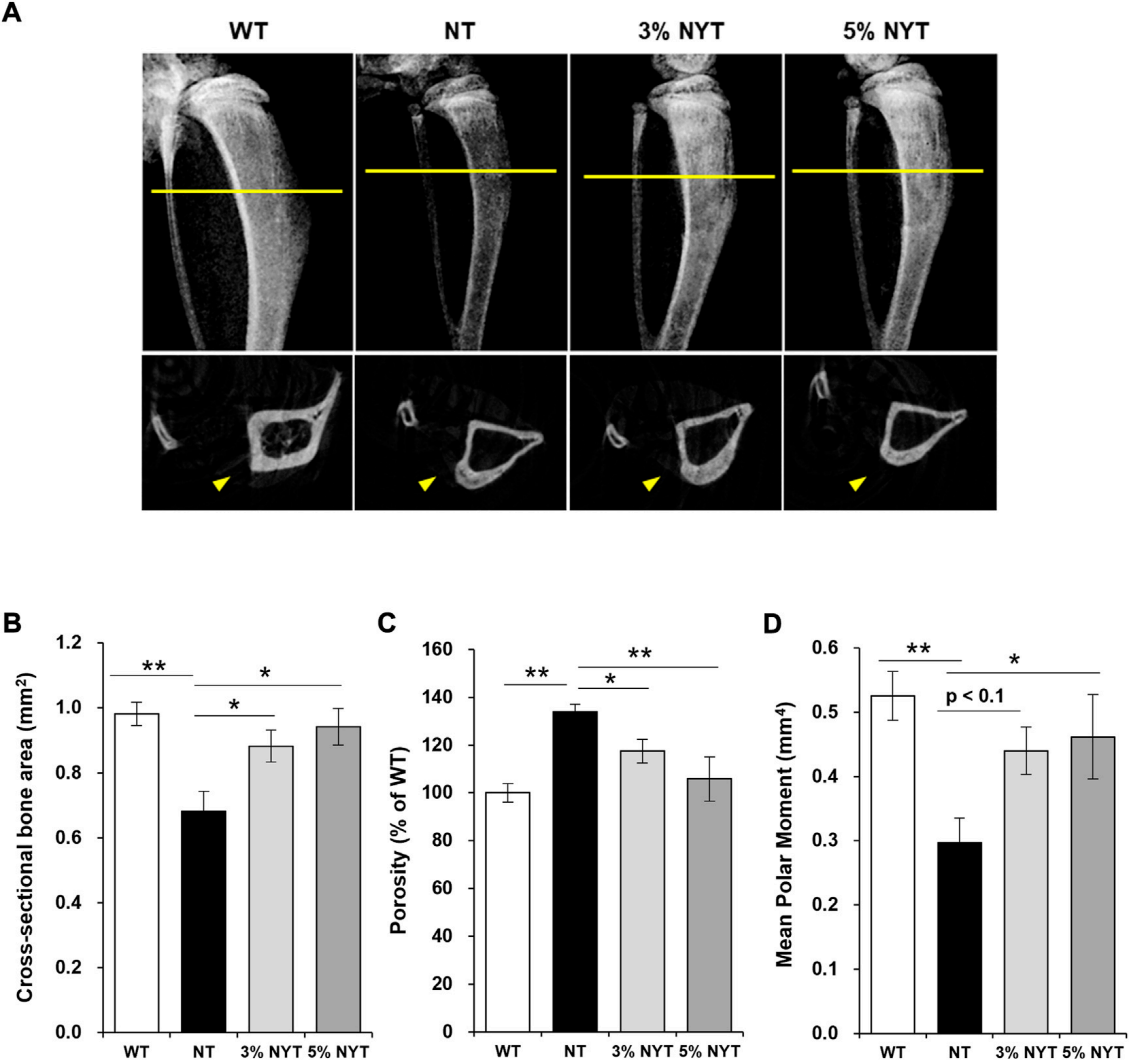
X-ray image of the dissected tibia of hindlimb (**A**, upper) and slice images of micro-CT scan (**A**, lower) on 59 days old are shown. The radiation absorption values of the both bones were analyzed by CT analyzer software, and determined the Cross sectional bone area **(B)** and Porosity **(C)** and Mean Polar Moment **(D)** of cortical bone. Data are indicated as mean and SEM values. Significant differences are presented on histograms: [*p < 0.05, **p < 0.01, Dunnett’s contrast, WT (*n* = 4), NT (8), 3% NYT (6), 5% NYT (4)].

The kl/kl mice have been reported to exhibit a pathology similar to osteoporosis in the central portion of bone, with a high degree of calcium deposition at the bone ends, similar to marble disease (Kuro-o et al., 1997). An X-ray image of tibia scanned by micro-CT is shown in Figure 5A. For Cross sectional bone area of cortical bone, the NT group showed a 30.58% decrease compared to WT group (*p* = 0.006) (Figure 5B). In contrast, the 3% NYT group showed a 10.15% reduction (*p* = 0.040), and the 5% NYT group showed only a 4.08% reduction (*p* = 0.017). Cortical bone porosity (%) increased 34.02% in the NT group compared to WT (*p* < 0.001), but only increased 17.47% and 5.83% in the 3% NYT and 5% NYT groups, respectively, showing a significant improvement over the NT group (*p* = 0.045 and 0.002) (Figure 5C). Mean Polar Moment of Inertia of cortical bone decreased 43.54% in the NT group compared to WT (*p* < 0.005), but only decreased 12.19% in the 5% NYT group, representing a significant improvement compared to the NT group (*p* = 0.047) (Figure 5D).

Trabecular bone showed no significant difference in tissue volume between the WT and NT groups. No significant difference in trabecular separation of cancellous bone was seen between WT and NT groups, but the 5% NYT group showed a significant decrease of 34.18% compared to the NT group (*p* = 0.038). These results suggest that NYT treatment is effective in maintaining cortical bone thickness and strength in kl/kl mice.

### Improvement in the skeletal muscular system with NYT treatment

**FIGURE 6:**
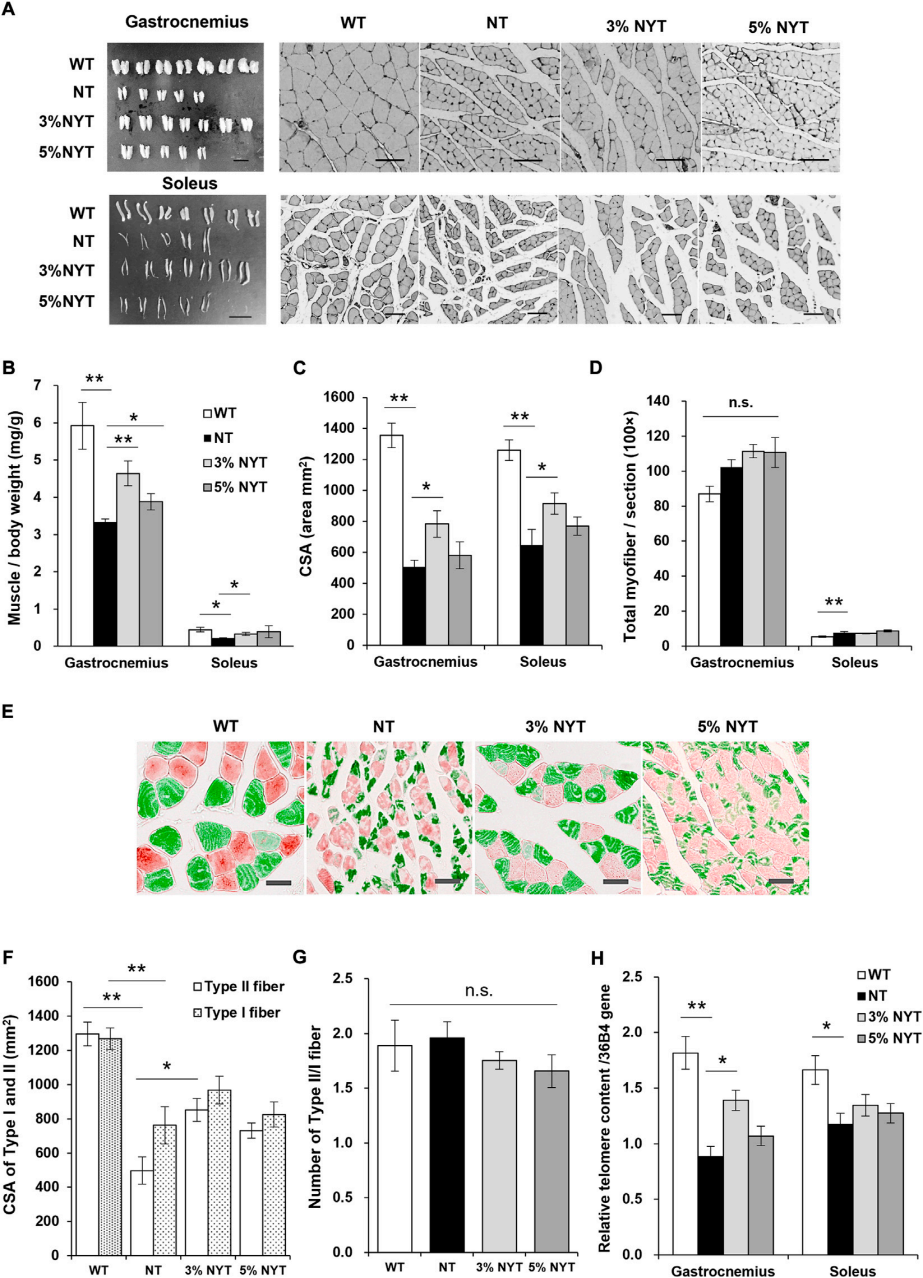
Effect of NYT on atrophy of fast myofiber fiber and its aging. The dissected triceps muscle, consisting of the gastrocnemius (**A**, upper left) and soleus muscles (**A**, lower left) and its wet weight **(B)** in 59 days, and histological immuno-staining (dystrophin) of muscle fibers (**A**, right) are shown, scale bar indicated 10 mm (left) and 50 μm (right). The CSA **(C)** and total count of myofiber of each muscle section **(D)** is shown. Images of fast type myofiber (green) and slow type myofiber (red) of soleus muscle stained by immunohistochemistry are shown in **(E)**, scale bar indicated 30 μm. The CSA of fast type or slow type myofiber **(F)** and total count of each myofiber **(G)** is shown. Relative telomere content of gastrocnemius and soleus muscles was measured using qPCR **(H)**. Data are plotted as mean and SEM values. Significant differences are presented on histograms: (*p < 0.05, **p < 0.01, Steel test **(B)** and Dunnett’s contrast **(C**–**H)**, WT (*n* = 7), NT (5), 3%NYT (7), 5%NYT (5).

The triceps surae muscle of the hind limbs comprises the of soleus and gastrocnemius muscles, and its activity indirectly controls step length and gait velocity (Honeine et al., 2013; Honeine et al., 2014). Wet weight of the gastrocnemius and soleus muscles are shown in Figure 6B. In the gastrocnemius muscle, muscle weight per body weight of the NT group decreased by 43.72% compared with that in the WT group (*p* = 0.012), whereas the 3% NYT group was maintained at 21.61% decrease, and were significantly different compared with the NT group, respectively (*p* = 0.031). In a similar manner, in soleus muscle, the NT group showed a 52.48% decrease in the muscle weight compared with the WT group (*p* = 0.012), whereas the 3% NYT group was maintained at 26.19% decrease, and were significantly different from that in the NT group, respectively (*p* = 0.048). A linear correlation exists between muscle fiber thickness and muscle strength, and as Cross sectional area (CSA) decreases with age, muscle strength also decreases. Anti-dystrophin stained images of each muscle are shown in Figure 6A, and CSAs of gastrocnemius and soleus muscle are shown in Figure 6C. In the NT group, CSA was reduced 62.62% and 48.66% in gastrocnemius and soleus muscle compared with that in the WT group (*p* < 0.001, each), whereas the 3% NYT groups maintained at 42.23% and 27.41% decrease, and were significantly different compared with the NT group, respectively (*p* = 0.017 and 0.041). The total number of muscle fibers per muscle section is also shown in Figure 6D. Gastrocnemius muscle showed no significant difference in number of muscle fibers among all groups. In contrast, soleus muscle showed a significant (*p* = 0.009) increase of 40.03% in the NT group compared to the WT, but no significant difference in the NYT group.

To evaluate the effect of NYT on slow (type I) and fast (type II) skeletal myofiber, soleus muscle, which contains a large number of both types, was stained using antibodies that specifically recognize slow/fast skeletal myosin heavy chain, as shown in Figure 6E. In the NT group, fast myofiber atrophied about 61.58% compared to the WT group (*p* < 0.001), while the 3%NYT group showed significantly only about 34.27% atrophy, making a significant difference compared to the NT group (*p* = 0.002) In contrast, the myofiber also atrophied about 39.92% in the NT group compared to the WT group (*p* < 0.001), but NYT treatment resulted in no significant difference (Figure 6F). Sarcopenia and disuse atrophy in the muscles of the elderly also reportedly alter the ratio of fast to slow muscle fibers, which changes the properties of the muscle as a whole, even if the muscles remain of the same thickness. The fast/slow myofiber composition ratio was therefore measured (Figure 6G). However, no significant changes were found among any of the groups (*p* = 0.687).

### NYT suppressed the aging of the gastrocnemius muscle, which is predominantly fast myofiber

Telomere content in genomic DNA of the muscles is shown in Figure 6H. Telomere content of gastrocnemius and soleus muscles in the NT group was reduced significantly, by 51.39% and 29.51%, as compared to that in the WT group (*p* < 0.001 and 0.008). In contrast, the 3%NYT groups maintained at 23.52% reduced, and were significantly different compared to that in NT group (*p* = 0.009).

### Effect of NYT against oxidative stressors in the gastrocnemius muscle

To evaluate the impact of oxidative stressor accumulation on muscle atrophy in kl/kl mice and the effect of NYT, the indicator of the oxidative stressor, 8-OHdG, was detected in sections of gastrocnemius muscle. The results showed that 8-OhdG positive nuclei increased by 342.81% in NT as compared with that in the WT group (*p* < 0.001), whereas the 3% NYT group maintained at 99.20% increased, significantly (*p* < 0.001, Supplementary Figure S3).

### Effect of NYT treatment on skeletal muscle protein synthesis and degradation systems in skeletal muscle

**FIGURE 7:**
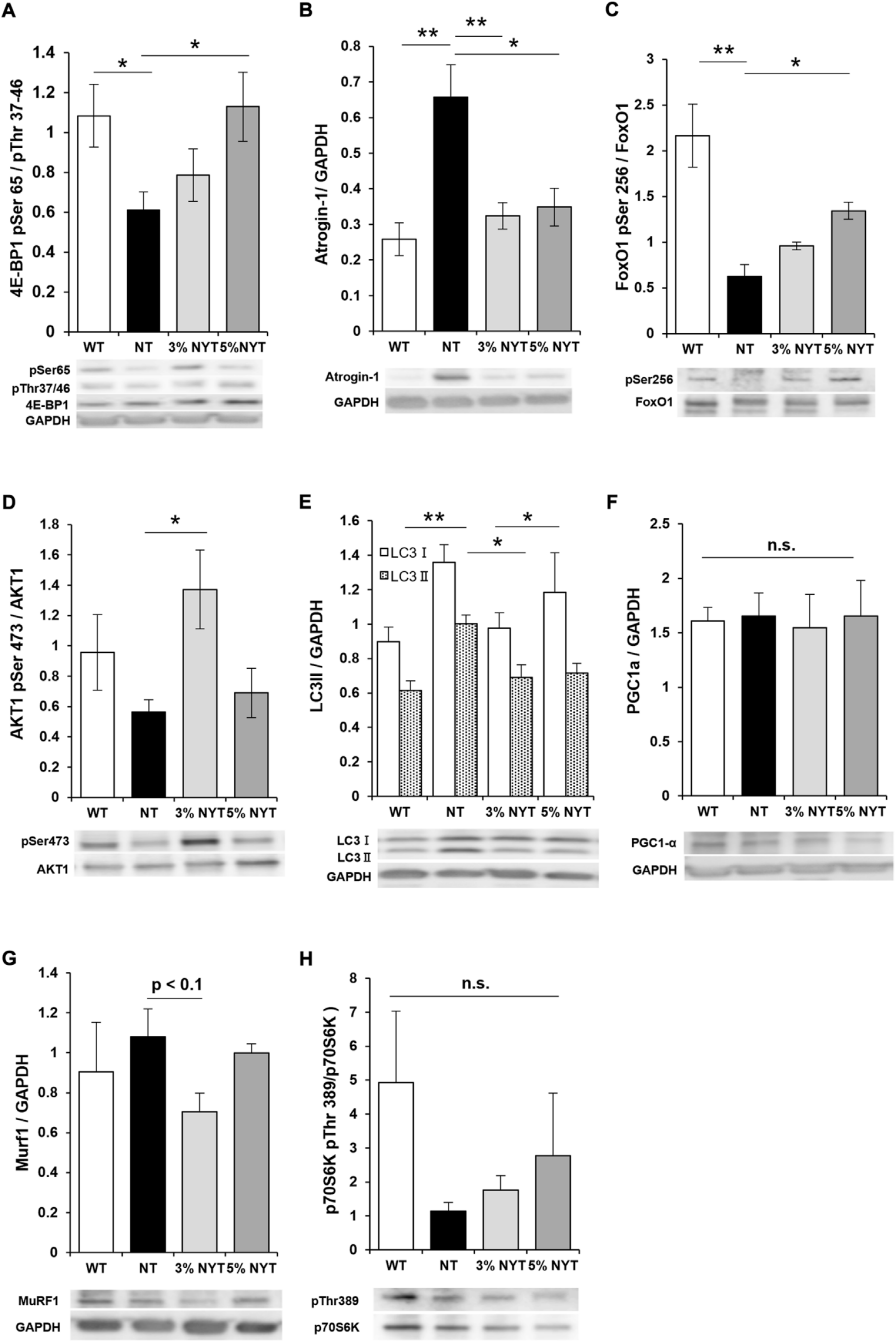
The activities of myoprotein synthesis factors and myoproteolytic factors in 59 days old were evaluated by western-blotting analysis using the dissected gastrocnemius muscle. The detected bands are shown below the graph of quantified arbitrary unit. Factors related to muscle protein synthesis: phosphorylation of AKT1 at Ser473 **(D)**, phosphorylation of 4E-BP1 at Ser65/Thr37-46 **(A)**, phosphorylation of p70S6K at Thr389 **(H)**, the amount of PGC1α **(F)**; degradation-related factors: the amount of Atrogin-1 **(B)**, the amount of Murf1 **(G)**, phosphorylation of FoxO1 Ser256 **(C)**, the amount of LC3II **(E)**. Data are indicated as mean and SEM values. Significant differences are presented on histograms: [*p < 0.05, **p < 0.01, Dunnett’s contrast, WT (*n* = 5), NT (8), 3% NYT (6), 5% NYT (4)].

To evaluate the effects of NYT on muscle protein synthesis or myodegradation processes, factors involved in synthesis of muscle proteins (multiple phosphorylation of 4E-BP1, phosphorylation of AKT1, expression of PGC1α, and phosphorylation of p70S6K) and factors involved in induction of muscle protein degradation and their regulators (expression of Atrogin-1 and Murif1, change to LC3II, dephosphorylating of FoxO1) were evaluated in gastrocnemius muscle in which differences in muscle mass were observed between NT and NYT treatments (Figures 7A–H). Multiple phosphorylation of 4E-BP1 decreased by 43.73% in the NT group compared to the WT group (*p* = 0.041), whereas the 5% NYT group showed an additional 4.21% significant increase from the WT (*p* = 0.036). In contrast, no significant changes were seen in PGC1α or p70S6K in any groups. In addition, atrogin-1, one of the E3 ubiquitin ligase that induces muscle protein degradation, was increased by 154.45% compared to WT (*p* = 0.002), but only 25.09% increase in the 3% NYT group compared to the NT group (*p* = 0.005), and only a 34.91% increase in the 5% NYT group. (*p* = 0.025) Also, phosphorylation of Ser256 in FoxO1, which interferes with atrogin-1 activity, was reduced by 71.14% in the NT group compared to the WT (*p* = 0.009), but only significantly by 37.90% in the 5% NYT group (*p* = 0.018). In addition, phosphorylation of Ser473 of AKT1 was no significant difference in NT group compared to wild type, but 43.17% increase significantly in 3% NYT group (*p* = 0.011). In contrast, Murf1, another E3 ligase under the control of FoxO1 showed no significant change in all groups, but a trend toward a decrease in the 3%NYT group compared to the NT group. In addition, when the activity of autophagy, another mechanism that induces proteolysis of muscle protein, was evaluated in terms of LC3 protein status, LC3II increased 63.31% in the NT group compared to the WT (*p* = 0.001), while in the 3% NYT and 5% NYT group it increased only 12.68% and 16.87% increase, significantly (*p* = 0.006 and 0.029).

### Improvement in walking performance with NYT treatment

**FIGURE 8:**
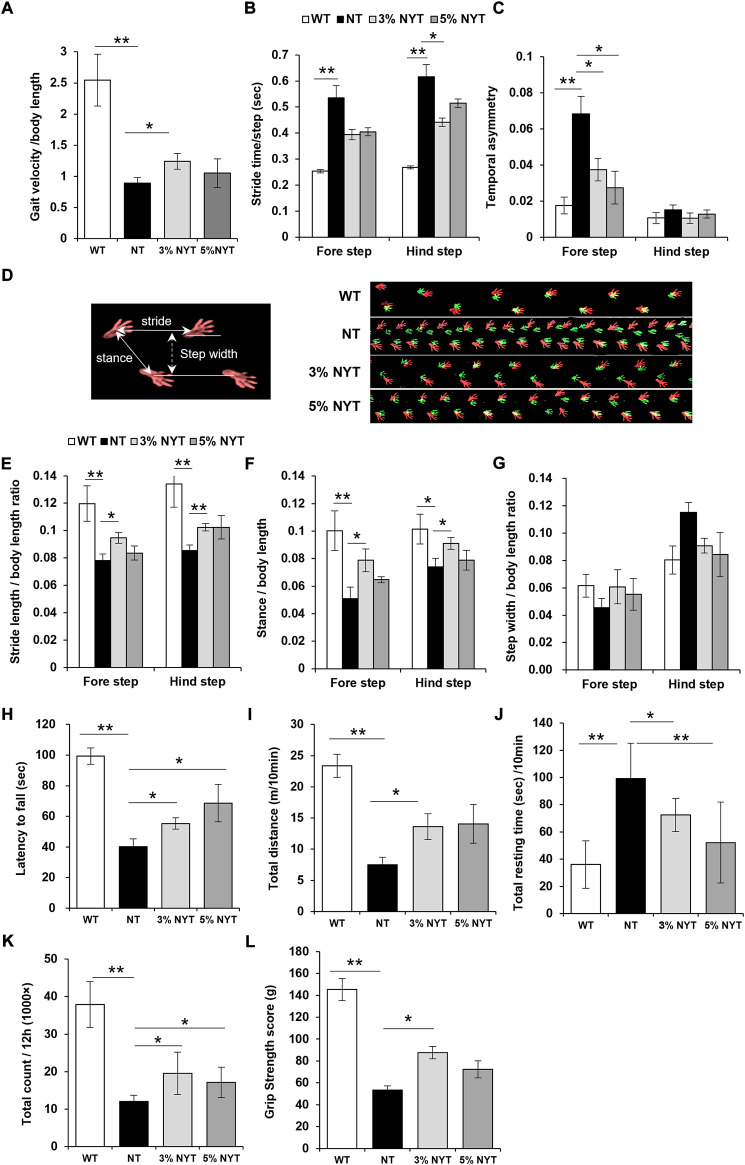
Effects of NYT on impairment of physical performance with aging. At 57 days of age, mice were subjected to the free walking test on the walking path, and evaluated their temporal parameters of gait: velocity **(A)**, stride time **(B)**, and temporal asymmetry **(C)**, and spatial parameters of gait: stride length **(E)**, stance length **(F)**, step width **(G)** using the footprint images (**D**, right panel) of the fore step (green) and hind step (red). Spatial gait parameters are indicated by the white arrow (**D**, left panel). Performance of latency on the rotarod from accelerated cylinder (4–20 rpm/3 min) is shown in **(H)**. Spontaneous locomotor activity was measured using the distance travelled **(I)** and resting time **(J)** for 10 min in the open field, and total counts of locomotion in the breeding cage for 12 h during the nocturnal period **(K)**. Measuring the Grip strength were performed using the force meter. The highest peak tension of the released their grip from the mesh in four trials were shown in **(L)**. Data are indicated as mean and SEM values. Significant differences are presented on histograms: (*p < 0.05, **p < 0.01, Steel test **(A**,**B**,**E**,**F**,**H**,**I**,**K**,**L)** and Dunnett’s contrast **(C**,**G**,**J)**, WT (*n* = 6), NT (7), 3%NYT (6), 5%NYT (5).

Gait velocity at 58 days old is shown in Figure 8A. The NT group showed a marked decrease of 64.82% in gait velocity compared with the WT group (*p* = 0.007), while the 3% NYT group was maintained at 51.17% decrease, and were significantly different compared to the NT group (*p* = 0.041). To investigate factors influencing the gait velocity, detailed gait parameters were analyzed as described previously (Mendes et al., 2015). Stride time is the time for the leg of the mouse to kick the ground, then land, and then kick again (Figure 8B). In the fore-legs, stride time of the NT group was increased by 111.51% as compared to the WT group (*p* < 0.001), while no significant difference was seen between NYT treatment groups. In a similar manner, in the hind leg, the NT group showed a 129.76% increase in sride time as compared to the WT group (*p* < 0.001), while the 3%NYT group was maintained at 64.65% increase, and were significantly different compared to the NT group (*p* = 0.048). The rate of temporary asymmetry refers to the time balance between right and left leg motions (Figure 8C). In the fore-legs, asymmetry index of the NT group was increased by 287.15% as compared to the WT group (*p* < 0.001) while the 3%NYT and 5% NYT group was maintained at 112.02% and 55.79% increase, and were significantly different compared to the NT group (*p* = 0.026 and 0.005). In several trials in the NT group, mice appeared to fall temporarily, and a marked increase was noted in the asymmetry index. However, mice walked smoothly thereafter, and these data were excluded from the data analyses. Gait velocity correlates with survival in older adults (Studenski et al., 2011). Thus, these data were evaluated using the Spearman correlation coefficient to evaluate whether this model can be applied. The correlation coefficient was 0.723 (*p* = 0.000213) in all kl/kl mice and 0.743 (*p* = 0.0345) in the 3% NYT group, with a strong correlation between gait velocity and survival rate (Supplementary Figure S1).

Footprints of steps are shown in Figure 8D. Data on stride length are shown in Figure 8E. In forelegs steps, stride length of the NT group decreased by 34.78% compared with the WT group (*p* = 0.002), while the 3%NYT group was maintained at 20.98% decrease, and were significantly different compared to the NT group (*p* = 0.003). In a similar manner, in hindleg steps, the NT group showed a 36.18% decrease in stride length compared with the WT group (*p* = 0.002) while the 3%NYT group was maintained at 23.55% decrease, and were significantly different compared to the NT group (*p* = 0.001). Data on stance length are shown in Figure 8F. In foreleg steps, stance length of the NT group decreased by 49.17% compared with the WT group (*p* < 0.001) while the 3%NYT group was maintained at 21.44% decrease, and were significantly different compared to the NT group (*p* = 0.009). In a similar manner, in hindleg steps, the NT group showed a 27.18% decrease in stance length compared with the WT group (*p* = 0.024) while the 3%NYT group was maintained at 10.26% decrease, and were significantly different compared to the NT group (*p* = 0.024). Data on step width are shown in Figure 8G. But, no significant changes were found among all groups.

### Improvement in smooth walking ability with NYT treatment

Data on latency to fall from the rotarod test are shown in Figure 8H. A 59.61% reduction in retention time was seen in the NT group as compared to the WT group (*p* < 0.001), while the 3% NYT and 5% NYT group was maintained at 44.33% and 30.97% reduction, and were significantly different compared to the NT group (*p* = 0.002 and *p* = 0.007).

### Improvement in the spontaneous motor activity with NYT treatment

In the short-term motor activity test in the open field, total distance was reduced by 67.77% in the NT group as compared to the WT group (*p* = 0.001) (Figure 8I) while the 3%NYT group was maintained at 41.79% reduction, and were significantly different compared to the NT group (*p* = 0.041). Resting time was increased by 175.64% in the NT group as compared to the WT group (*p* < 0.001), while the 5%NYT group was maintained at 44.99% increase, and were significantly different compared to the NT group (*p* = 0.002) (Figure 8J). In the long-term motor activity test, total counts of locomotion during the nocturnal period were measured using a Supermex apparatus on the breeding cage. Total counts were reduced by 68.27% in the NT group as compared to the WT group (*p* = 0.006) while the 3% NYT and 5% NYT group was maintained at 48.47% and 54.79% reduction, and were significantly different compared to the NT group (*p* = 0.027 and *p* = 0.042) (Figure 8K).

### Improvement in the grip strength with NYT treatment

Fast-twitch muscle fibers are thought to be used when great instantaneous force is required, and slow-twitch muscles fibers are those used when endurance is required. NYT may be effective for maintaining fast twitch myofiber. To test this hypothesis, the grip strength was evaluated as a test of instantaneous force. Maximal grip strength at day 58 is shown in Figure 8L. A 63.22% decrease in strength was seen in the NT group compared to the WT group (*p* = 0.001). In contrast, the decrease in the grip strength in the 3% NYT group was maintained at 39.66%, significantly different from that in the NT group (*p* = 0.011).

## Discussion

Mutation of the klotho gene causes multiple premature aging phenotypes, including a shortened lifespan in mice (Kuro-o et al., 1997), and some SNPs in the human α-klotho gene are associated with reduced lifespan. We found that NYT prolonged survival, improved a number of age-related diseases and motor dysfunction in kl/kl mice as a model of human aging-related frailty.

Unlike the other organs, the heart does not atrophy with age. Conversely, even in normotensive subjects, left ventricular mass progressively increases throughout life, reaching its greatest magnitude in senescence (Lie and Hammond, 1988; Lernfelt et al., 1991; Paciaroni and Fraticelli, 1995). Left ventricular hypertrophy is considered a pathological response to increased blood pressure and cardiac workload and is associated with increased risks of cardiovascular disease events and mortality (Levy et al., 1990; Vakili et al., 2001). Compared with WT mice, kl/kl mice manifested a significantly increased ratio of cardiac weight standardized to total body weight, increased relative left ventricular wall thickness, increased CSA area of individual cardiomyocytes, and changes in gene expression that are characteristic of left ventricular hypertrophy (LVH) (Faul et al., 2011). Our results suggest that NYT treatment prevented age-related cardiac hypertrophy in kl/kl mice in a dose-dependent manner.

There is abundant evidence of shared features between pulmonary emphysema and aging lung (MacNee and Tuder, 2009). Pulmonary emphysema is a devastating disease in which the alveolar walls are destroyed and enlarged, reducing the surface area for gas exchange and limiting the elastic recoil of the lungs (Guest, 1998). The first histological emphysematous changes in kl/kl mice appear at 4 weeks old with both destruction of alveolar walls and air space enlargement (Suga et al., 2000). Our findings indicate that treatment with 3% NYT may suppress age-related emphysema-like symptoms in kl/kl mice.

One of the most acknowledged changes of the aging immune system is regression, or involution of the thymus (George and Ritter, 1996; Taub and Longo, 2005; Lynch et al., 2009). Atrophy of the thymus is widely observed during aging of both humans and mice (Kuro-o et al., 1997). Age-associated thymic involution involves a decrease in tissue mass and cellularity, together with a loss of tissue organization with the net outcome being a reduction in T cell output (George and Ritter, 1996; Taub and Longo, 2005; Lynch et al., 2009). The altered thymic activity with aging is a key trigger toward declining immune function in the aged (Aw and Palmer, 2011). In kl/kl mice, the thymus was normal in size at earlier developmental stages, but was barely detectable in any kl/kl mice at 6–9 weeks old (Kuro-o et al., 1997). In the study, 3% NYT suppressed atrophy of the thymus and tended to suppress splenic atrophy in kl/kl mice. Moreover, NYT improved the cellular populations of lymphocytes and monocytes. These results suggest that NYT improved immunosenescence. The major facets of immunosenescence include persistent low-grade inflammation (“inflammaging”), decreased abilities to fight infections or cancers, impaired ability to efficiently respond to new antigens, an increased incidence of autoimmune phenomena, and impaired wound repair (Goronzy and Weyand, 2013; Sadighi Akha, 2018).

High incidence of testicular atrophy and hypogonadism are currently attracting wide attention due to the global aging of the population and environmental pollution (McBride et al., 2016; Di Ciaula and Portincasa, 2020). The aging process for the testes eventually leads to abnormal steroidogenesis, reduced spermatogenesis, and decreased semen quality (Liao et al., 2016; Jeremy et al., 2019). In male kl/kl mice, the testes are atrophied and mature spermatozoa are not evident (Kuro-o et al., 1997). In the 3% NYT group, atrophy of the testes was significantly suppressed and sperm were present. These results suggested that NYT may have improved gonadal function in kl/kl mice.

Osteoporosis is a common aging-related disease characterized by decreased bone mass and fragility fractures, and places a huge burden on society (Hu and Wang, 2022). The kl/kl mice exhibit a state of low turnover involving formation and resorption of bone, similar to humans (Kuro-o et al., 1997). In addition, the thickness of cortical bone is particularly reduced in kl/kl mice (Kuro-o et al., 1997). Our results in this study suggest that NYT treatment prevented age-related osteoporosis in kl/kl mice in a dose-dependent manner.

The maintenance of muscle mass and CSA were observed in the 60-day-old 3% NYT treatment group, consistent with recently reported clinical data (Sakisaka et al., 2018). The results of immunoblotting in the gastrocnemius of kl/kl mice suggests that NYT induced activation of 4E-BP1 in the protein synthesis system and regulated the pathway leading from FoxO1 to Atrogin1 in the protein degradation system. In addition, the ingredient or crude drug component of NYT that prevents skeletal muscle atrophy has been previously reported: extracts of *Paeonia lactiflora*, Citrus unshiu peel, and *Glycyrrhiza* radix prevent inflammation-induced muscle atrophy (Kim et al., 2016; Bae et al., 2020); Schisandra fruit, catalpol of Rehmanniae radix, and panaxatriol of Panax Ginseng stimulate muscle protein synthesis after exercise by activating PGC-1 (Kim et al., 2014; Li et al., 2014) or AKT/mTORC1 signaling pathway and muscle glucose disposal (Hou et al., 2015; Takamura et al., 2017); and atractylenolide III of Atractylodes rhizome reduce muscle wasting by attenuating disease-derived oxidative stress (Wang et al., 2019). These components may thus contribute to reduced muscle atrophy with aging. To consider the reasons for improvements in muscle atrophy, an important factors is that body weight and spontaneous motor activity were maintained in the 3% NYT group, but are reduced in the terminal stage of kl/kl mice.

In disuse syndrome in the elderly, free radicals are generated in muscle tissue and induce muscle proteolysis, resulting in skeletal muscle atrophy (Powers et al., 2007; Venturelli et al., 2014). Elevated levels of free radicals are also the main cause of shortening of the telomere length in skeletal muscle, representing a marker of aging and attenuation of mobility (Cawthon et al., 2003; Østhus et al., 2012; Rubinek and Modan-Moses, 2016; Stenbäck et al., 2019). In highly proliferative tissue, shortening of telomere length occurs due to the replication process, but occurs in skeletal muscle due to free radical exposure (Venturelli et al., 2014). In this study, the telomere content of muscles was maintained in the 3% NYT group as compared to that in the NT. Improvements in spontaneous locomotion with NYT are considered to suppress the generation of free radicals. Drugs with antioxidant activity also suppress disuse muscle atrophy by scavenging free radicals (Powers, 2014). Orally administered NYT scavenges the free radicals *in vivo* (Egashira, 1999; Egashira et al., 2003). In the study, 8-OHdG positive nuclei were significantly increased in NT compared to WT, while a significant improvement was observed at 3% NYT. Therefore, as in previous reports, NYT has the ability to scavenge oxidative stressors or inhibit the production of its, and the improvement in muscle atrophy in the present study is related to this effect.

In the gait test, gait velocity and various spatial-temporal parameters of mice in the terminal phase were significantly improved in the 3% NYT group as compared to the NT group. In previous studies on the relationship between aging and gait parameters, age and decreases in gait velocity, stride length, and stance length were strongly correlated in the elderly (Himann et al., 1988; Prince et al., 1997; Samson et al., 2001). Decreased stride length in kl/kl mice has also been reported (Kuro-o et al., 1997). Moreover, step width and toe-out angle (progression foot angle) increased to stabilize balance during low-speed walking or to ameliorate atrophy of the hip joint muscles in the elderly (Miyatsuji et al., 2007; Shimada et al., 2010). The latency to fall in the rotarod test evaluates gait parameters that cannot be evaluated with free walking and that are known to decline with age (Tung et al., 2016). All or some of these parameters improved following 3% NYT treatment, resulting in improved gait velocity.

Ataxia is caused by declines in the physical functions of various body parts, such as skeletal muscle and bone, CNS, respiratory organs, and sensory system. Aging not only causes degenerative loss of muscle mass and strength, termed sarcopenia, but also is closely associated with decreased mobility (Momoki et al., 2017). In particular, in the natural attenuation of gait performance due to aging, decreased strength and atrophy of the lower limb muscles are important factors that prevent normal walking performance (Toda et al., 2016; Song and Geyer, 2018; Uematsu et al., 2018). In kl/kl mice, some lower limbs muscle is significantly reduced compared to the WT (Phelps et al., 2013).

Weight loss without increased mobility causes muscle wasting. Generally, annual weight loss of over 5% in older adults indicates progression of sarcopenia and frailty with muscle loss (Fried et al., 2001; Bales and Ritchie, 2002). In this study, kl/kl mice had decreased mobility and lost 15.2% body weight in 6 days. Furthermore, decreased locomotor activity is often observed in frail elderly and easily causes disuse muscle atrophy (Miller et al., 2019). Muscle atrophy in kl/kl mice was caused by growth hormone depletion and poor muscle regeneration (Balkovic and Hsiung, 1985; Rubinek and Modan-Moses, 2016; Sahu et al., 2018), but we considered that exacerbation of muscle atrophy involved disuse muscle atrophy (Kuro-o et al., 1997; Wall et al., 2013; Eren et al., 2014).

To accurately control the drug dosage of kampo medicine, it is reasonable to use forced oral administration. However, in our preliminary study, forced oral administration caused significant stress to the terminal stages of kl/kl mice, with an increase in the number of animals dying the day after administration. Therefore, we administered the NYT diet. To determine the dosage, we administered 1%, 3%, and 5% NYT in a preliminary study. The 1% NYT group showed no change trend from NT in survival rate, so the 3% and 5% concentrations were selected. In addition, in our previous study on NYT for disuse muscle atrophy (Takemoto et al., 2021), a 5% NYT concentration of mixed feed was more effective than 3%, and other studies have reported that 3% NYT was effective in maintaining muscle and alveolar integrity in COPD Model Mice (Miyamoto et al., 2020). The kl/kl mouse is a model that exhibits emphysema, which also led us to select these two doses.

The 5% NYT group was less effective than the 3% NYT group in various tests in the current study, so the 5% concentration is presumably not the optimal concentration to administer in male kl/kl. But, in this experiment, the change over time in body weight of the 5% NYT group was not different from that of the NT group or showed an increasing trend, and spontaneous locomotion and survival rates were similar, suggesting that the 5% NYT concentration is not optimal but is not toxic. In contrast, in our previous study of disuse muscle atrophy of C57BL/6, which is a background of kl/kl, a 5% NYT concentration was more effective than 3% NYT concentration (Takemoto et al., 2021). Which concentration is the optimal treatment dose against the frailty? There are some hypotheses for this question. For example, it is possible that some tissues may have different active ingredients than others and that the same ingredients may have different tissue deliverability, as well as the amount of ingredients needed. However, verification of these hypotheses will require many additional analyses, including identification of the active ingredient and measurement of tissue deliverability, and will be the subject of future research.

NYT is designed to be taken orally in human adult at a daily dose of 6700 mg, divided into 2 or 3 doses per day. Because kl/kl mice are housed with wild-type protected mice, it was not possible to measure the exact amount of food intake, but presuming that it is approximately 1 g/day, which is the intake of an individual of the same body weight of other lines, the human daily dose corresponds to approximately 2.5% NYT. However, it is possible that the amount of ingredients transferred into the body per hour may differ between the twice-daily dosing and the intermittent dietary administration, and it is also possible that as the amount of food ingested decreases with aging, the actual amount administered may have also changed.

## Conclusion

Survival was prolonged in the 3% NYT group, and age-related histological declines in heart, lung, thymus, testis, bone tissue, muscles and age-related motor dysfunction were improved in the 3% NYT group. In addition, a strong correlation was observed between gait velocity and survival in all kl/kl mice and the 3% NYT group. These results suggest that 3% NYT is more effective for kl/kl mice. The mechanisms for prolongation of kl/kl mice survival that were previously reported include attenuation of senescence factor (Eren et al., 2014; Sato et al., 2015; Fujitsuka et al., 2016) and improvements in calcium homeostasis (Nabeshima, 2006; Voelkl et al., 2013; Nabeshima et al., 2014; Leibrock et al., 2015). Further investigation is required to understand the mechanisms underlying improved survival. NYT has the potential to make a significant contribution to extending healthy lifespan.

## Data Availability

The original contributions presented in the study are included in the article/Supplementary Materials, further inquiries can be directed to the corresponding author.

## Supplementary material

Supplementary Figure S1 Evaluation of leukocyte population. The counts of leukocyte population of peripheral blood were shown in painted bar graphs. Samples were analyzed as follow: peripheral blood were blocked with Trustain FcX (biolegend) and then stained with anti-mouse CD45-APC, CD3e-PerCP-Cy5.5, CD19-PE, CD49b-FITC, and ZombieNIR-dye (biolegend), followed by hemolysis with Versalyse (Beckman Coulter) and fixed. The population was assayed using a CytoFlex Flow cytometer (Beckman). The gating was performed as follows: granulocytes were identified with CD45+ SSChigh, lymphocytes with CD45high SSC-Alow, and monocytes with SSC-Amid CD45high. Among lymphocytes, T cells were identified with CD3e+, B cells with CD19+, and NK cells with CD49b+. Data are indicated as mean and SEM values.

Supplementary Figure S2 There is a correlation between survival period and walking speed. Plot of correlation between survival period and walking speed of kl/kl at the age of 57 days. Spearman correlation coefficient is 0.723 (*p* = 0.000213) in all groups and 0.743 (*p* = 0.0345) in 3% NYT.

Supplementary Figure S3 Effect of NYT against oxidative stressors in the gastrocnemius muscle. The oxidative stress indicator 8-OHdG production was evaluated in gastrocnemius muscle at 59 days of age by histological immunostaining of muscle nuclei (red, 8-OHdG, top panels), and total nuclei (blue, hematoxylin) are shown, scale bar indicated 80 μm. The ratio of 8-OHdG-positive nuclei/total nuclei is plotted as the mean, and SEM values are shown (under panel). Significant differences are presented on histograms [***p* < 0.01, Dunnett’s contrast, WT (*n* = 5), NT (7), 3%NYT (6).
