# Supplementary figures and images for: Impact of Ninjin’yoeito on frailty and short life in klotho-hypomorphic (kl/kl) mice
Source: Front Pharmacol. 2022 Oct 24;13:973897. doi: 10.3389/fphar.2022.973897 (PMC9637981; doi:10.3389/fphar.2022.973897)

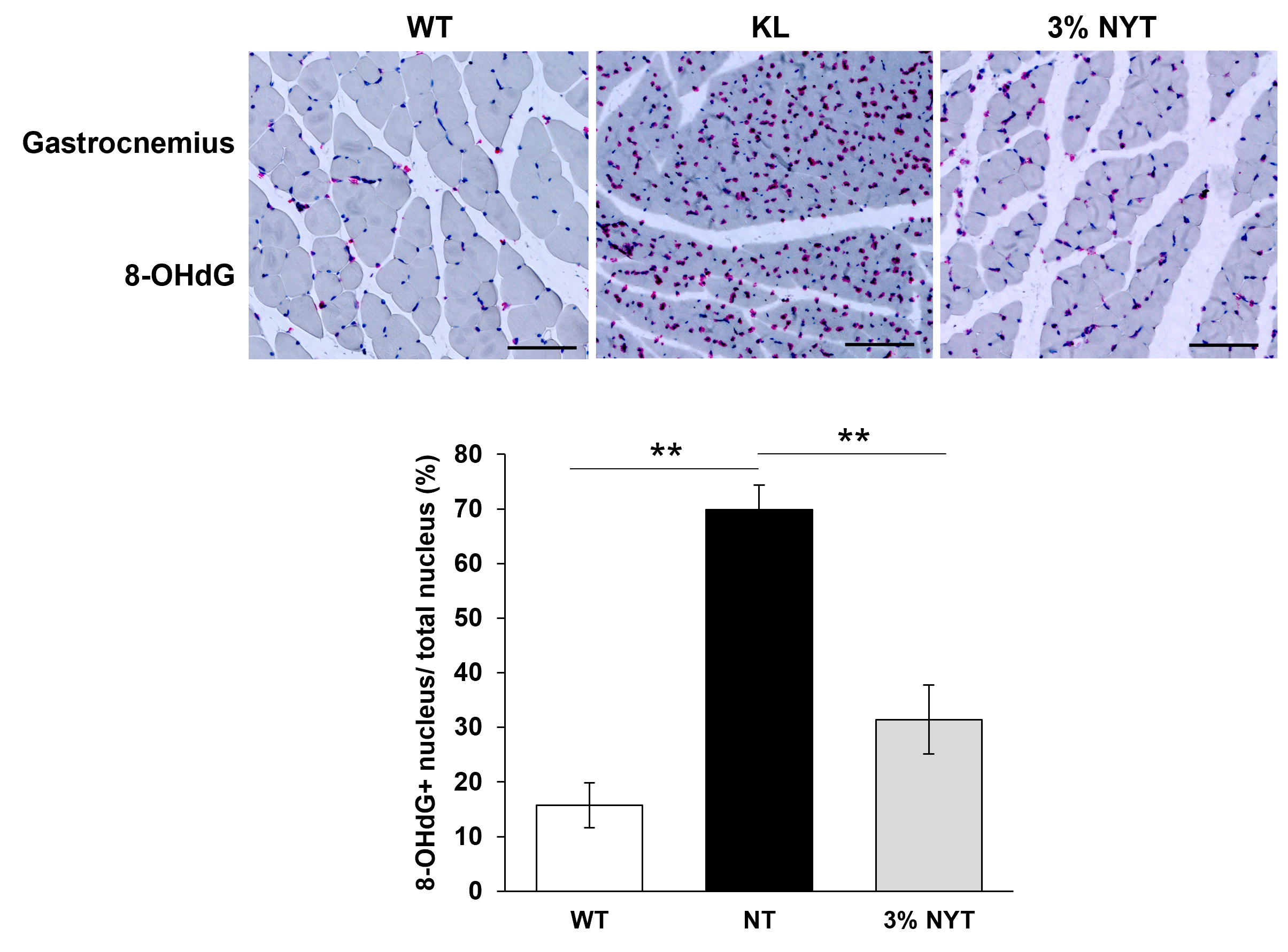

Supplement: Supplementary file 1 [file Image3.TIF]

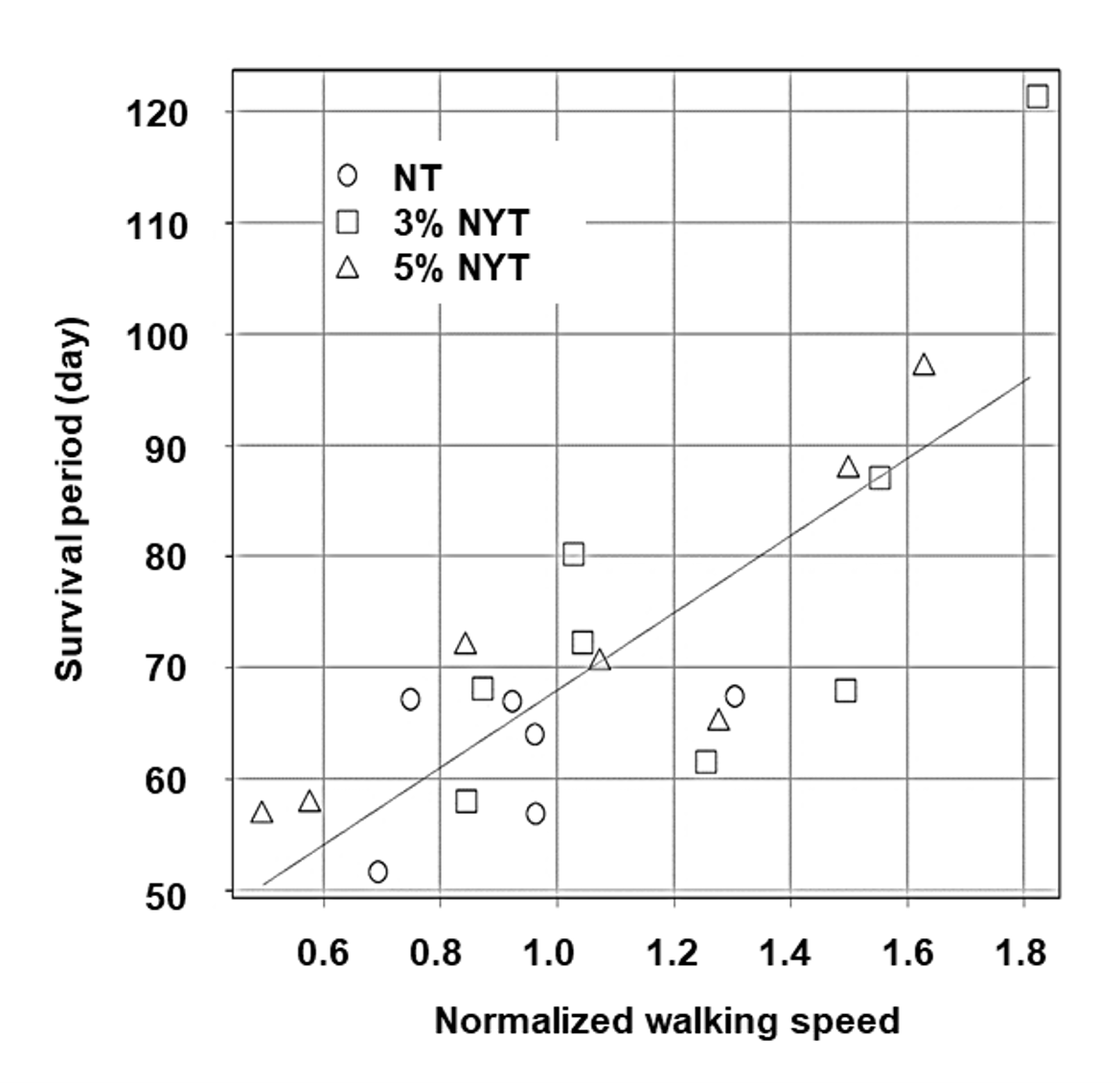

Supplement: Supplementary file 2 [file Image2.TIF]

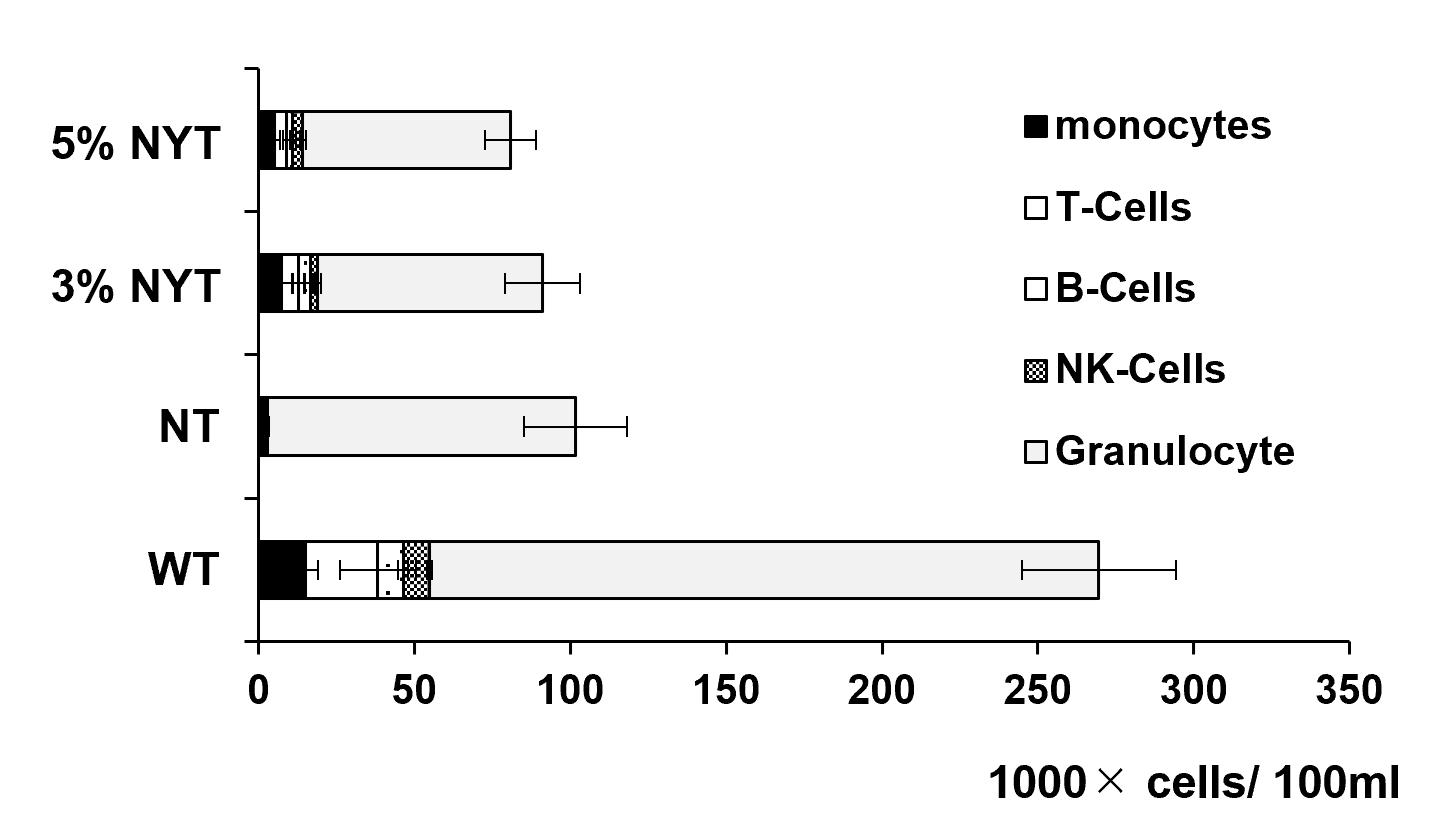

Supplement: Supplementary file 3 [file Image1.TIF]
